# Associations between apathy and global cognition in a 5‐year follow‐up of individuals with Alzheimer's disease

**DOI:** 10.1111/jnp.70044

**Published:** 2026-03-30

**Authors:** Henna‐Riikka Loukola, Maikki Kärkkäinen, Tuomas Selander, Kirsi Honkalampi, Anne M. Koivisto, Toni T. Saari

**Affiliations:** ^1^ School of Educational Sciences and Psychology University of Eastern Finland Joensuu Finland; ^2^ Science Services Center Kuopio University Hospital, Wellbeing Services County of North Savo Kuopio Finland; ^3^ NeuroCenter, Neurology Kuopio University Hospital Kuopio Finland; ^4^ Departments of Geriatrics and Neurology, Helsinki University Hospital and Department of Neurosciences, Faculty of Medicine University of Helsinki Helsinki Finland; ^5^ Institute for Molecular Medicine Finland, HiLIFE University of Helsinki Helsinki Finland; ^6^ Present address: School Social Worker and Psychologist Services Wellbeing Services County of Central Finland Jyväskylä Finland; ^7^ Present address: Psychiatric Outpatient Clinic, Hospital Nova Wellbeing Services County of Central Finland Jyväskylä Finland

**Keywords:** Alzheimer's disease, apathy, CERAD, cognition, dementia, longitudinal study

## Abstract

Apathy is a common neuropsychiatric symptom in Alzheimer's disease (AD) associated with cognitive and functional impairment. Longitudinal studies have examined the associations between apathy and cognition in AD and other dementias, but more information is needed to understand whether the relationships are consistent longitudinally, and whether apathy relates to decline in AD‐related cognitive domains or to drop‐out rates in the follow‐up. We used data from 236 people (age M = 75.15 and 51.3% female at baseline) with very mild or mild AD from the Finnish ALSOVA study with up to 5 years of follow‐up. Global cognition was measured with the Consortium to Establish a Registry for Alzheimer's disease‐Neuropsychological Battery, apathy with the Neuropsychiatric Inventory, and disease severity with the Clinical Dementia Rating‐Sum of Boxes. Associations between cognition and apathy were examined with correlation analyses, linear regression analyses and linear mixed models. In longitudinal analyses, we found that apathy was associated with worse global cognition (*B* = −0.39, SE = 0.12, *p* = .001) after adjusting for disease severity. However, apathy and global cognition were not consistently associated with one another in cross‐sectional analyses. Older age (OR = 0.95, *p* = .02), but not apathy, was associated with a lower likelihood of participating in the final follow‐up visit. In conclusion, apathy associates with worse global cognition longitudinally, but when examined cross‐sectionally, the apathy‐cognition associations are inconsistent. Apathy at baseline does not seem to affect drop‐out rates in a long follow‐up of individuals with very mild or mild AD.

## INTRODUCTION

Alzheimer's disease (AD) is the most common cause of dementia and a major burden to patients, their caregivers and aging societies (Nichols et al., 2022). In mild AD, a notable decline in episodic memory and other cognitive domains can be observed. Neuropsychiatric symptoms (NPSs) start to occur gradually, the most common ones being apathy, depression and increased irritability (Zhao et al., 2016). Apathy is the most common NPS in AD (Nobis & Husain, 2018), and its prevalence increases with disease progression (Grossman et al., 2021; Starkstein, 2006). Apathy is linked to worse functional performance, cognitive impairment and caregiver distress (Grossman et al., 2021; Lanctôt et al., 2017; Saari et al., 2020). Moreover, apathy has been found to increase the risk of conversion from normal cognitive aging to mild cognitive impairment (MCI; Fan et al., 2021; Geda et al., 2014; Hu et al., 2020) and from MCI to AD (Palmer et al., 2010; Richard et al., 2012; Roberto et al., 2021; Ruthirakuhan et al., 2019; Vicini Chilovi et al., 2009).

While there are some longitudinal studies investigating the associations between apathy and cognition in AD (Eikelboom et al., 2021; Starkstein, 2006) or dementia with Lewy bodies (Breitve et al., 2018), more research is needed using composite measures of tests assessing AD‐relevant cognitive domains. Previous studies in AD have often used the Mini‐Mental State Examination (MMSE; Folstein et al., 1975) as a measure of cognitive performance. While the MMSE is a clinically relevant measure for monitoring disease progression in AD, it has been shown that the Consortium to Establish a Registry for AD‐Neuropsychological Battery (CERAD‐NB; Morris et al., 1989; Welsh et al., 1994) total score can detect more granular differences in disease progression (Hallikainen et al., 2013). The superior performance of the CERAD‐NB over MMSE likely relates to sensitive detection of earliest AD‐related changes in memory and language, and to a lesser extent, executive functions. In contrast, the MMSE was not developed for assessing AD‐related changes (Chandler et al., 2005). Moreover, composite scores, such as the CERAD‐NB total score, have higher reliabilities than individual tests (Calamia et al., 2013).

Additionally, as NPSs are known to fluctuate over short and long intervals (Eikelboom et al., 2021, 2022; Vik‐Mo et al., 2018), examining the associations between apathy and cognition at different time points is also warranted. In general, associations between psychological constructs tend to be relatively weak in cross‐sectional settings, whereas aggregating data over multiple timepoints increases the strength of these associations (Epstein, 1980; Rushton et al., 1983), presumably due to reduced measurement error. Therefore, it is possible that owing to factors contributing to measurement error in NPS, such as caregiver burden (Pfeifer et al., 2013), the associations between cognition and apathy may be different when examined cross‐sectionally versus in aggregate over multiple timepoints.

There is a lack of comprehensive studies examining the associations between apathy and AD‐sensitive cognitive measures at various stages of the disease and across long, clinically relevant follow‐ups. To address this gap, the first aim of our exploratory study was to investigate the relationship between apathy and cognition in persons with AD across a 5‐year follow‐up. The second aim was to study whether the associations between apathy and cognition vary at different time points. Finally, we examined whether apathy and other clinical‐demographic factors predicted attrition in the follow‐up.

## MATERIALS AND METHODS

### Participants and study design

This study is part of the ALSOVA follow‐up study, which was conducted at the University of Eastern Finland and Kuopio University Hospital. The main purpose of the ALSOVA study was to examine how psychosocial interventions affect the institutionalization of patients with AD (Koivisto et al., 2016). Data consists of baseline measurements (2002–2006) and five annual follow‐up measurements, the most recent of which was carried out in 2011. All patients participating in the study underwent laboratory and neuropsychological tests as part of the diagnostic clinical evaluation. In addition, the evaluation included a brain MRI or CT scan. AD was diagnosed according to DSM‐IV (American Psychiatric Association, 1994) and NINCDS‐ADRDA criteria (McKhann et al., 1984).

A total of 236 patients and their caregivers participated in the study, with five patient—caregiver dyads dropping out due to the absence of AD, moderate AD and severely impaired vision. Inclusion criteria were very mild (Clinical Dementia Rating [CDR; Hughes et al., 1982] 0.5) or mild AD (CDR 1), residing in the community and having access to a caregiver (Hongisto, 2017). In addition, the patient had to be fluent in Finnish and could not have any comorbid conditions affecting cognition.

### Measures

CERAD‐NB (Morris et al., 1989; Welsh et al., 1994) was used to assess global cognition in this study. CERAD‐NB has been found to be a valid method to assess cognitive decline related to AD (Hallikainen et al., 2013), and the CERAD‐NB total score has been reported to be a valid method to assess the progression of AD (Rossetti et al., 2010). The Finnish version of the CERAD‐NB includes nine subtests, which evaluate different domains of cognition: Verbal Fluency (Isaacs & Kennie, 1973) assesses language functions and semantic memory, Abbreviated Boston Naming Test (Kaplan et al., 1978; Mack et al., 1992) assesses language functions, Word List Learning, Recall and Recognition tests (Morris et al., 1989) assess verbal memory and verbal learning, Constructional Praxis (Rosen et al., 1984) assesses visuoconstructive skills, Constructional Praxis Recall (Fillenbaum et al., 2011) assesses visual memory and Clock Drawing Test (e.g., Hazan et al., 2018) assesses executive and visuoconstructive functions. In addition, CERAD‐NB includes the Mini‐Mental State Examination (MMSE; Folstein et al., 1975), which is used to assess general cognitive impairment.

CERAD‐NB subtests, excluding Clock Drawing Test and MMSE, form the CERAD‐NB total score (0–100), where a lower score implies weaker cognitive functioning. In this study, the CERAD‐NB total score was used as a measure of global cognition.

The Neuropsychiatric Inventory (NPI; Cummings et al., 1994) was used to assess NPSs. The NPI includes informant ratings of delusions, hallucinations, agitation, anxiety, depression, apathy, irritability, euphoria, disinhibition, aberrant motor behaviour, sleep disorders and appetite disturbances. In this study, we used NPI apathy scores (0–12; scores 5, 7, 10 and 11 are not possible outcomes), which included frequency (1–4) and severity (1–3) of apathy. The total score is calculated by multiplying frequency (1–4) and severity (1–3) scores. In the total score, zero refers to persons who do not show symptoms of apathy, and a higher total score indicates more severe and/or frequent symptoms.

CDR was used to evaluate the severity of AD. In CDR, functional and cognitive abilities are evaluated with a semi‐structured interview of the patient and the patient's caregiver. Functional and cognitive abilities assessed in CDR include memory, orientation, judgement and problem solving, community affairs, home and hobbies and personal care. These domains are evaluated with a five‐point scale, and these evaluations form the overall rating where CDR 0 = no dementia, CDR 0.5 = questionable dementia, CDR 1 = mild cognitive impairment, CDR 2 = moderate cognitive impairment, CDR 3 = severe cognitive impairment. More refined Clinical Dementia Rating Sum of Boxes (CDR‐SOB) scores can be defined by the sum of the six domain scores (O'Bryant, 2008). CDR‐SOB scores range from 0 to 18 and can be used to evaluate functional—cognitive status: 0 refers to normal outcome, 0.5–4.0 questionable cognitive impairment, 4.5–9 mild dementia, 9.5–15.5 moderate dementia and 16.0–18.0 severe dementia (O'Bryant, 2008).

### Statistical methods

Descriptive statistics (means, standard deviations, percentages) of the demographic variables (gender, age at baseline, years of education), CERAD‐NB total scores, NPI apathy scores and CDR‐SOB scores were calculated. Distributions of the variables were examined by histograms, Kolmogorov–Smirnov test and skewness and kurtosis values. Spearman's rank‐order correlation was used to examine the relationship between CERAD‐NB total scores and NPI apathy scores in different measurement points.

Linear mixed models (LMMs) were used to examine the association between apathy and cognition, aggregating data over the 5‐year follow‐up. This way, missing data at some point does not remove the case from the entire analytic sample, and the maximal amount of data is used. The assumption of normally distributed residuals was checked with histograms. In the LMM analysis, the CERAD‐NB total score was set as the dependent variable, whereas demographic variables, CDR‐SOB score and NPI apathy scores were set as fixed effects. Random intercepts for participants and an unstructured covariance structure for the repeated measures were used. At first, univariate analyses were used to examine the relationship between NPI apathy scores and CERAD‐NB total scores, when other independent variables were not included in the model. Multivariate analysis was used to assess the relationship between NPI apathy scores and CERAD‐NB total scores when age, sex, years of education and CDR‐SOB scores were included in the model. Variables with the highest p‐values were removed stepwise so that only statistically significant variables explaining CERAD‐NB total scores remained. Interactions with apathy and CDR‐SOB were also examined; sensitivity analyses with data stratified by CDR global score categories would follow in case of a significant interaction. Another sensitivity analysis used a subset of participants who completed at least some of the measurements on the fifth follow‐up. Linear regression analyses were used to examine the associations between apathy and demographic variables with the CERAD‐NB total score; additional models were run with CDR‐SOB.

Factors relating to drop‐out were examined with logistic regression analysis. Namely, we examined whether CERAD‐NB total score, NPI apathy, CDR‐SOB, age, sex or years of education at baseline were related to participating in the fifth and final follow‐up visits of this study. For the analysis, we created a dichotomic variable for participation vs. drop‐out at the fifth follow‐up visit (0 = did not participate, 1 = participated) and used this variable as the dependent variable.

Statistical analyses were performed using IBM SPSS version 27 (IBM Corp, 2020). The threshold of statistical significance was set to *p* < .05. R version 4.5.1 (R Core Team, 2025) was used for the figure.

### Ethical considerations

ALSOVA follow‐up study complies with the Declaration of Helsinki. The ALSOVA study was approved by the Kuopio University Hospital ethics committee (64/00), the Finnish Supervisory Authority for Welfare and Health and the Finnish Ministry of Social Affairs and Health. Patients and caregivers participating in the study were given information both orally and in writing. Volunteered participants signed an informed consent form, and they had the right to withdraw from the study.

## RESULTS

### Descriptive statistics

Descriptive statistics of the participants are presented in Table 1. At baseline (*n* = 236), the percentage of females was 51.3%, whereas at the fifth follow‐up, the percentage increased to 55.4%. Mean participant age decreased from 75.15 to 73.62. At baseline, the mean years of education was 7.58, whereas at the fifth follow‐up, the mean increased to 8.03. 162 participants (68.6%) dropped out during the study.

**TABLE 1:** Demographic and clinical characteristics of the study sample.

	Baseline	Follow‐up 1	Follow‐up 2	Follow‐up 3	Follow‐up 4	Follow‐up 5
*n*	236	198	168	132	85	74
Age, baseline, M (SD)	75.15 (6.52)	74.98 (6.77)	74.95 (6.89)	74.69 (6.78)	74.12 (6.57)	73.62 (6.39)
Education years, M (SD)	7.58 (3.29)	7.64 (3.39)	7.64 (3.32)	7.67 (3.26)	7.55 (3.33)	8.03 (3.34)
Female, *n* (%)	121 (51.3)	105 (53.0)	91 (54.2)	72 (54.5)	47 (55.3)	41 (55.4)
CDR‐SOB, *n*	236	198	168	128	83	73
CDR‐SOB, M (SD)	4.14 (1.47)	5.64 (2.34)	7.07 (2.98)	8.32 (3.37)	9.25 (3.66)	10.77 (4.17)
CDR global rating, *n* (%)						
0.5	128 (54.2)	64 (32.3)	27 (16.1)	8 (6.3)	4 (4.8)	2 (2.7)
1	108 (45.8)	112 (56.6)	97 (57.7)	74 (57.8)	38 (45.8)	30 (41.1)
2	0 (0)	22 (11.1)	42 (25)	40 (31.3)	35 (42.2)	28 (38.4)
3	0 (0)	0 (0)	2 (1)	6 (4.7)	6 (7.2)	13 (17.8)
CERAD‐NB total score, *n*	234	194	163	125	76	63
CERAD‐NB total score, M (SD)	51.58 (11.85)	46.90 (13.01)	42.09 (14.77)	39.49 (15.74)	37.83 (15.76)	36.78 (18.18)
NPI apathy, *n*	236	197	168	132	82	73
NPI apathy, M (SD)	1.50 (2.01)	1.86 (2.22)	2.31 (2.42)	3.00 (3.13)	3.52 (3.18)	3.62 (3.26)

Abbreviations: CDR‐SOB, Clinical Dementia Rating‐Sum of Boxes; CERAD‐NB, Consortium to Establish a Registry for Alzheimer's Disease‐Neuropsychological Battery; NPI, Neuropsychiatric Inventory.

**FIGURE 1:**
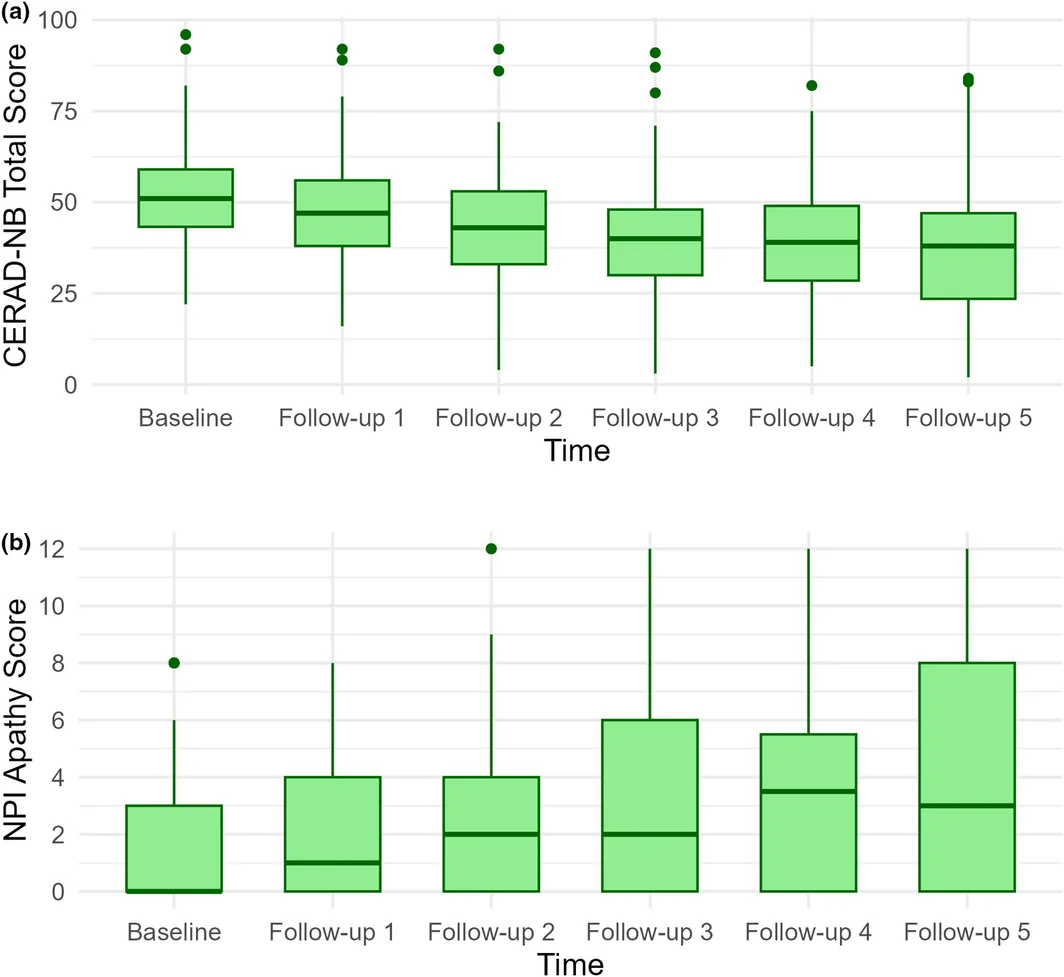
Box‐and‐whisker plots of cognitive and apathy scores over time. (A) CERAD‐NB total scores at different measurement points. (B) NPI apathy scores at different measurement points. Bold horizontal lines indicate median, and hinges (or lower and upper boundaries of the boxes) indicate the first and third quantiles, respectively. Whiskers (or vertical lines) indicate the largest and smallest observations equal to or lesser (upper whisker) or greater (lower whisker) than the 1.5 interquartile range from the respective hinges. Dots outside the whiskers represent outliers. CERAD‐NB, Consortium to Establish a Registry for Alzheimer's Disease‐Neuropsychological Battery; NPI, Neuropsychiatric Inventory.

The mean CDR‐SOB score of the patients increased from 4.14 (*n* = 236) to 10.77 (*n* = 73) during the study period. At baseline, the mean global cognition was 51.58 (*n* = 234) and the mean NPI apathy score was 1.50, whereas at the fifth follow‐up, the mean global cognition was 36.78 (*n* = 63) and the mean NPI apathy score was 3.62 (*n* = 73). The developments of the CERAD‐NB total score and NPI apathy scores over time are shown in Figure 1. Overall, the standard deviations of CDR‐SOB scores, global cognition and apathy increased during the study period.

### Correlations between global cognition and apathy

The correlations between global cognition and apathy are presented in Table 2. Apathy correlated significantly with global cognition at the second follow‐up (*r*_s_ = −.20) and the fifth follow‐up (*r*_s_ = −.41). Correlations of global cognition at different measurement points were significant (*r*_s_ = .62–.91). Similarly, apathy scores at different time points correlated significantly with each other (*r*_s_ = .25–.63), except for the correlation between baseline and fifth follow‐up visit.

**TABLE 2:** Correlations between CERAD‐NB total score and NPI apathy at different time points.

	Baseline	Follow‐up 1	Follow‐up 2	Follow‐up 3	Follow‐up 4	Follow‐up 5
Baseline	−.01	.77**	.65**	.72**	.62**	.72**
Follow‐up 1	.39**	−0.11	.83**	.83**	.64**	.74**
Follow‐up 2	.42**	.51**	−.20**	.82**	.71**	.79**
Follow‐up 3	.26**	.35**	.49**	−.13	.84**	.81**
Follow‐up 4	.30**	.45**	.43**	.63**	−.14	.91**
Follow‐up 5	0.19	.33**	.25*	.48**	.53**	−.41**

*Note*: The diagonal shows cross‐sectional correlations between CERAD‐NB and apathy. The upper triangle shows correlations between CERAD‐NB measurements at different time points and the lower triangle shows correlations between apathy scores at different time points.

Abbreviations: CERAD‐NB, Consortium to Establish a Registry for Alzheimer's Disease‐Neuropsychological Battery‐Neuropsychological Battery; NPI, Neuropsychiatric Inventory.

** Significant at *p* < .01.

* Significant at *p* < .05.

### Association between apathy and cognition in the 5‐year follow‐up

In the univariate LMM analyses, apathy (*B* = −0.50, SE = 0.12, *p* < .001), years of education (*B* = 1.11, SE = 0.22, *p* < .001) and CDR‐SOB scores (*B* = −2.48, SE = 0.10, *p* < .001) were statistically significantly associated with global cognition when analysed in aggregate over the 5‐year follow‐up (Table 3). In the multivariate analysis, apathy (*B* = −0.39, SE = 0.12, *p* = .001), years of education (*B* = 0.71, SE = 0.20, *p* < .001) and CDR‐SOB scores were associated with global cognition (Table 3). In separate models, interactions for CDR‐SOB and apathy (*p* = .864) and CDR global score and apathy (*p* = .163) were not significant and CDR‐stratified analyses were not pursued. A sensitivity analysis using data from participants who participated in the fifth follow‐up supported the overall results: apathy (*B* = −0.36, SE = 0.17, *p* = .31), years of education (*B* = 2.0, SE = 0.33, *p* < .001) and CDR‐SOB (−2.17, SE = 0.15, *p* < .001) associated with global cognition.

**TABLE 3:** Results of linear mixed models of NPI apathy, CDR‐SOB and covariates predicting the CERAD‐NB total score.

	Univariate			Multivariate		
	*B*	SE	*p*	*B*	SE	*p*
Sex						
Male	0.39	1.52	.797			
Age, baseline	−0.11	0.12	.345			
Education years	1.11	0.22	**<.001**	0.71	0.20	**<.001**
CDR‐SOB	−2.48	0.10	**<.001**	−2.38	0.10	**<.001**
NPI apathy	−0.50	0.12	**<.001**	−0.39	0.12	.**001**

*Note*: Bold values are significant at *p* < .05.

Abbreviations: CERAD‐NB, Consortium to Establish a Registry for Alzheimer's Disease‐Neuropsychological Battery; CDR‐SOB, Clinical Dementia Rating‐Sum of Boxes; NPI, Neuropsychiatric Inventory.

### Association between apathy and cognition in different measurement points

Out of sex, age, years of education and apathy, apathy and years of education were the only variables that were significantly associated with global cognition in cross‐sectional analyses (Table 4). At baseline (*F*_4,229_ = 7.170, *p* < .001, *R*_*a*_^*2*^ = .096) and the first (*F*_4,188_ = 3.970, *p* = .004, *R*_*a*_^*2*^ = .058) and the third follow‐up (*F*_4,120_ = 5.511, *p* < .001, *R*_*a*_^*2*^ = .127), the only variable significantly related to global cognition was years of education, after Bonferroni correction. Both apathy and years of education were significantly related to global cognition at the second (*F*_4,158_ = 4.629, *p* = .001, *R*_*a*_^*2*^ = .082) and the fifth follow‐up (*F*_4,57_ = 9.367, *p* < .001, *R*_*a*_^*2*^ = .354). At the fourth follow‐up (*F*_4,69_ = 1.203, *p* = .317, *R*_*a*_^*2*^ = .011), none of the variables were significantly related to global cognition.

**TABLE 4:** Cross‐sectional linear regression analysis results of NPI apathy, CDR‐SOB and covariates predicting the CERAD‐NB total score.

	Baseline			Follow‐up 1			Follow‐up 2		
	*B*	95% CI	*p*	*B*	95% CI	*p*	*B*	95% CI	*p*
*Baseline through follow‐up 2*									
Intercept	38.96	19.52, 58.39	<.001*	48	25.32, 70.69	<.001*	29.92	2.27, 57.58	.**034**
Sex^a^	−0.92	−3.85, 2.01	.538	0.1	−3.58, 3.78	.959	−0.26	−4.74, 4.23	.911
Age, baseline	0.06	−0.18, 0.29	.647	−0.08	−0.36, 0.20	.558	0.1	−0.23, 0.44	.542
Education years	1.2	0.74, 1.67	<.001*	0.85	0.30, 1.39	.003*	0.99	0.29, 1.70	.006*
NPI apathy	−0.11	−0.85, 0.63	.771	−0.71	−1.54, 0.13	.095	−1.37	−2.36, −0.38	.007*

	Follow‐up 3			Follow‐up 4			Follow‐up 5		
	*B*	95% CI	*p*	*B*	95% CI	*p*	*B*	95% CI	*p*
*Follow‐up 3 through follow‐up 5*									
Intercept	44.55	8.95, 80.15	.**015**	27.88	−19.30, 75.06	.242	38.97	−12.17, 90.12	.133
Sex^a^	−1.33	−6.65, 3.99	.622	0.39	−7.05, 7.82	.918	−5.41	−12.99, 2.18	.159
Age, baseline	−0.18	−0.61, 0.25	.411	0.06	−0.52, 0.63	.849	−0.12	−0.73, 0.50	.711
Education years	1.55	0.70, 2.39	**<.001** *	1.05	−0.09, 2.18	.071	2.24	1.03, 3.44	**<.001** *
NPI apathy	−0.94	−1.81, −0.06	.**036**	−0.75	−1.96, 0.46	.221	−2.72	−3.92, −1.51	**<.001** *

*Note*: Bold values are significant at *p* < .05.

Abbreviations: CERAD‐NB, Consortium to Establish a Registry for Alzheimer's Disease‐Neuropsychological Battery; CDR‐SOB, Clinical Dementia Rating‐Sum of Boxes; NPI, Neuropsychiatric Inventory.

^a^ Reference = male.

* Significant after Bonferroni correction for multiple comparisons.

In addition, we examined whether apathy had an interaction with CDR‐SOB at any time point after adjusting for age, sex and years of education. Apathy (*p* = .131–.974) or the interaction variable of apathy and CDR‐SOB scores (*p* = .079–.784) were not significantly associated with global cognition at any measurement point. At baseline (*F*_6,227_ = 8.559, *p* < .001, *R*_*a*_^*2*^ = .163), and the third (*F*_6,117_ = 22.017, *p* < .001, *R*_*a*_^*2*^ = .506) and fifth follow‐up (*F*_6,55_ = 18.531, *p* < .001, *R*_*a*_^*2*^ = .633), years of education and CDR‐SOB scores were statistically significantly associated with global cognition, after Bonferroni correction. At the first (*F*_6,186_ = 16.810, *p* < .001, *R*_*a*_^*2*^ = .331), second (*F*_6,156_ = 20.163, *p* < .001, *R*_*a*_^*2*^ = .415) and fourth follow‐up (*F*_6,67_ = 10.217, *p* < .001, *R*_*a*_^*2*^ = .431), CDR‐SOB scores were significantly associated with global cognition.

### Drop‐out analysis

The results of the drop‐out analyses are presented in Table 5. The model correctly classified 70.1% of the participants. The Hosmer—Lemenshow test result was not statistically significant (*χ* ^2^(8) = 9.504, *p* = .302), indicating the model fits the data well. In brief, older age at baseline predicted later drop‐out of the study (OR = 0.95, 95% CI: 0.90–0.99, *p* = .02), while apathy (OR = 0.92, 95% CI: 0.79–1.08, *p* = .31), years of education (OR = 0.99, 95% CI: 0.90–1.09, *p* = .82), global cognition (OR = 1.02, 95% CI: 0.99–1.05, *p* = .12) and CDR‐SOB (OR = 0.88, 95% CI: 0.71–1.09, *p* = .25) were not related to dropping out of the study.

**TABLE 5:** Logistic regression analysis of associations between baseline factors and participating in the fifth follow‐up study visit (0 = no participation, 1 = participation).

	OR	95% CI	*p*
Sex^a^	0.86	0.48–1.54	.61
Age, baseline	0.95	0.90–0.99	.**02**
Education years	0.99	0.90–1.09	.82
NPI apathy	0.92	0.79–1.08	.31
CERAD‐NB total score	1.02	0.99–1.05	.12
CDR‐SOB	0.88	0.71–1.09	.25

*Note*: Bold values are significant at *p* < .05.

Abbreviations: CDR‐SOB, Clinical Dementia Rating‐Sum of Boxes; CERAD‐NB, Consortium to Establish a Registry for Alzheimer's Disease‐Neuropsychological Battery; CI, confidence interval; NPI, Neuropsychiatric Inventory; OR, odds ratio.

^a^ Reference = male.

## DISCUSSION

The aim of our study was to investigate the association between apathy and cognition at different time points and across a 5‐year follow‐up of individuals with clinical AD. We found a negative association between apathy and cognition in individuals with AD when examining the entire follow‐up data, but cross‐sectional associations were more varied. Our results are mostly in line with prior research, according to which there is a link between apathy and cognition (Palmer et al., 2010; Richard et al., 2012). The association between apathy and cognition is likely influenced by several factors.

First, apathy has a long history of being conceptualized as a multidimensional construct (Dickson & Husain, 2022; Miller et al., 2021; Saari, 2023), most definitions of which include some dimension that overlaps with executive functions. The classic definition of Levy and Dubois (2006) posits that cognitive apathy arises from executive dysfunction in the form of impaired planning and working memory, whereas affective‐emotional apathy reflects disturbances in reward processing and assigning emotional value to contexts, and the behavioural dimension refers to lack of self‐directed thought or behaviours with relatively preserved responses to external prompts. Following this three‐dimensional model, Kumfor et al. (2018) found that cognitive apathy was more severe than affective‐emotional or behavioural in AD. Affective‐emotional apathy has been found to be more prevalent in individuals with more than 5 years since symptom onset, signalling the importance of this apathy domain in more progressed AD (Wei et al., 2020). Considering these findings, the results of the present study could relate to the overlap of progressive executive dysfunction and an increase in the cognitive aspect of apathy. However, since the NPI was used as the apathy measure in this study, different domains of apathy and their relationships to cognition could not be teased out.

Second, in terms of neuroanatomical substrates, it has been observed that the same cortical areas are partly involved in cognitive apathy and planning ability in patients with Alzheimer's disease (Eggins et al., 2022), so it is possible that the connection between apathy and cognition can be partly explained by the same brain regions being affected (McPherson et al., 2002; Mehak et al., 2023; Rosenberg et al., 2015). However, it is worth noting that the evidence for the three‐dimensional nature of apathy is somewhat controversial (Dickson & Husain, 2022; Mehak et al., 2023). For example, some studies validating multidimensional scales for apathy find equally good or better support for one (rather than three) dimensions, or the behavioural and cognitive domains are not meaningfully separable from one another (Dickson & Husain, 2022; Radakovic et al., 2017). Since the NPI does not differentiate between different dimensions of apathy, it may be more conservative to interpret our findings as apathy as a unitary construct developing mostly in parallel with cognitive decline (Mehak et al., 2023). The most consistent associations of apathy as a unidimensional construct have been found with disturbances in the anterior cingulate–subcortical circuit (Bonelli & Cummings, 2007; Mehak et al., 2023; Rosenberg et al., 2015). The anterior cingulate cortex in particular has been associated with cognitive functions such as attention, working memory and cognitive control (Gasquoine, 2013). In addition to the anterior cingulate cortex, atrophy in the amygdala could also relate to decline in memory performance and global cognition with concomitant apathy (Stouffer et al., 2024).

In the second research question, we examined whether the association between apathy and cognition is heterogeneous across the follow‐up. We found an association between apathy and cognition only at the second and last follow‐up points after adjusting for covariates. The heterogeneity in associations between cognition and apathy across the follow‐up may relate to the previously discussed issue of the NPI not separating different types of apathy. Affective‐emotional and behavioural apathy have been associated with caregiver burden, but cognitive apathy has not (Kumfor et al., 2018); therefore, it is possible that the apathy scores are even more inflated by caregiver burden when the apathy profile is predominantly affective‐emotional or behavioural. Accordingly, the lack of associations at most stages of the study could arise from the NPI measure capturing behavioural and affective‐emotional apathy. These dimensions are considered separable from cognitive apathy, which in turn could manifest in worse CERAD‐NB scores through increased executive dysfunction (McPherson et al., 2002). Additionally, the standard deviations of the CERAD total scores, the NPI apathy total scores and the CDR‐SOB scores of the participants increase during the follow‐up, meaning that the participants differ more from each other in terms of cognition, apathy and the severity of dementia as the follow‐up progresses. Overall, apathy symptoms seem to increase as the follow‐up progresses, and the most significant change in the CDR‐SOB scores appears to occur between the fourth and fifth follow‐up points. Instead, cognition appears to decline the most between the first and second follow‐up points. On the other hand, a nearly equally significant change is observed in cognition and severity of dementia, for example, between the baseline and the first follow‐up measurement points, so no direct conclusions can be made regarding the impact of these changes.

The lack of consistent associations between apathy and cognition at every time point may also relate to our use of CERAD‐NB as a cognitive measure. As it is primarily used for detecting and monitoring AD‐related changes, it has a significant memory component. Eikelboom et al. (2021) found that apathy had significant cross‐sectional associations with the MMSE, but not with memory domain scores in individuals with AD. In another study, apathy was associated with cognitive tests measuring executive function but not with other cognitive tests in individuals with AD (McPherson et al., 2002). Moreover, the contrasting results between cross‐sectional and longitudinal analyses may relate to the well‐established finding that relationships between two psychological constructs tend to become stronger when aggregated over multiple time points as measurement error decreases (Rushton et al., 1983). Thus, the fluctuating nature of NPSs (Eikelboom et al., 2021, 2022; Vik‐Mo et al., 2018), including apathy, may be mitigated in a longitudinal setting aggregating data across time.

In the last research question, we wanted to investigate which factors are associated with the attrition observed in the study. Previous studies have shown that men have shorter survival than women after an AD diagnosis (Todd et al., 2013), which could manifest as a sex effect in drop‐out analyses. However, we found that only younger age at baseline was associated with participation at the fifth follow‐up measurement. Those who were older at baseline likely had a higher comorbid disease burden, which may have led to dropping out of the study. Similarly, spousal caregivers of older participants may have developed health issues that prevented the dyad from participating later in the study. Disease severity at baseline did not associate with the likelihood of participating in the fifth follow‐up assessment. However, as all participants had very mild or mild AD at baseline, it is possible that individuals with more rapid disease progression in the first few years of follow‐up dropped out from the final study visit, and this effect was not detected with our analysis focusing on baseline variables only. Interestingly, apathy did not appear to have a significant association with participating in the fifth follow‐up. The finding that baseline apathy was not associated with drop‐out in this study is encouraging for observing the long‐term effects of pharmacological and non‐pharmacological interventions for apathy in AD (Mortby et al., 2021).

Our study has some limitations that should be considered when interpreting the results. Since the patients included in the study had AD and a mean age of 75 years, attrition was quite high, which should especially be taken into account in the linear regression analyses. As the follow‐up progresses, the number of participants decreases, and for example, only 62 individuals are included in the analyses at the fifth follow‐up. This introduces certain limitations to the generalizability of the linear regression analyses at the later measurement points, and thus also to the interpretation of the second research question. Furthermore, the proportion of individuals in moderate to severe stages is notable in the later follow‐up visits, and it is possible that these individuals might have experienced sundowning and fluctuation of symptoms that could be reflected in some of the scores. The CDR and the NPI measure changed behaviours over longer periods of time, but conducting the CERAD‐NB at an inopportune time might have worsened performance in these cognitive tasks. We did not have data for the time of day for measurements and could not adjust for potential sundowning effects, which is a limitation of our study.

Additionally, the study took place in Finland, and the focus was on identifying individuals with very mild or mild AD. A limitation of this design is that identifying patients at this early stage may not be representative of when the diagnoses are usually given, and the timing and means of AD diagnosis vary noticeably by country. Therefore, our results may not generalize well to different healthcare systems. However, identifying individuals with AD in the early stages of dementia allowed us to have a markedly long follow‐up.

When examining the results, it is important to note that no direct conclusions can be drawn about causality. Therefore, it cannot be stated whether apathy affects cognition or vice versa. It is likely that apathy and cognition have bidirectional relationships: as apathy symptoms increase, a person's involvement in activities decreases, which in turn negatively affects cognition through decreased social and cognitive stimulation. Additionally, cognitive decline likely contributes to the increase in apathy symptoms, as a decline in cognition may reduce a person's willingness to engage in activities, and cognitive difficulties could also reduce participation in social situations.

Despite its limitations, our study has several strengths. The longitudinal setting allows for the examination of the relationship between apathy and cognition over an extended period and at different time points as AD symptoms progress. Another advantage is that the exclusion criteria for participation were other coexisting cognitive impairments. Therefore, our study provides insights into the relationship between apathy and cognition, specifically in clinical AD. Studying apathy in distinct neurodegenerative diseases rather than all‐cause dementia has been identified as a research priority (Massimo et al., 2018). The understanding of the biological continuum of AD has increased rapidly in recent years (e.g., Jack et al., 2024), which has shifted the focus to preclinical and prodromal states of AD. However, it is worth noting that individuals with AD and their family members also need information about what cognitive and NPSs to expect and how they develop during the disease. Therefore, increasing the understanding of AD symptoms and their relationships during disease progression is still needed.

Another strength of our study lies in the measures used. The CERAD‐NB provides a comprehensive assessment of AD‐related cognitive domains, and when examining subtle changes in cognition, the CERAD total score is more sensitive than the commonly used MMSE (Chandler et al., 2005; Paajanen et al., 2011). Including CDR in our analyses is also an advantage, as the prevalence of apathy increases with disease severity (Grossman et al., 2021; Starkstein, 2006) and failing to account for disease severity might overestimate the association between apathy and cognition. Future longitudinal studies should examine the associations of different dimensions of apathy with cognition once the temporal measurement invariance of such apathy scales is established.

## CONCLUSIONS

In conclusion, we found apathy to be associated with global cognition in individuals with AD across a follow‐up of up to 5 years. Cross‐sectional associations between apathy and global cognition were more heterogeneous, possibly owing to the fluctuating nature of NPSs. Apathy at baseline does not seem to affect drop‐out rates in a follow‐up of individuals with very mild or mild AD.

## AUTHOR CONTRIBUTIONS

**Henna‐Riikka Loukola:** Writing – original draft; writing – review and editing; formal analysis. **Maikki Kärkkäinen:** Writing – original draft; writing – review and editing; formal analysis. **Tuomas Selander:** Writing – review and editing; data curation. **Kirsi Honkalampi:** Writing – review and editing. **Anne M. Koivisto:** Resources; writing – review and editing; funding acquisition. **Toni T. Saari:** Conceptualization; writing – original draft; supervision; writing – review and editing.

## CONFLICT OF INTEREST STATEMENT

The authors report no conflicts of interest.

## Data Availability

Research data are not shared.
